# The cellular niche for intestinal stem cells: a team effort

**DOI:** 10.1186/s13619-020-00061-5

**Published:** 2021-01-01

**Authors:** Guoli Zhu, Jiulong Hu, Rongwen Xi

**Affiliations:** 1 National Institute of Biological Sciences, No. 7 Science Park Road, Zhongguancun Life Science Park, Beijing, 102206 China; 2 School of Life Sciences, Tsinghua University, Beijing, 100084 China; 3 Tsinghua Institute of Multidisciplinary Biomedical Research, Tsinghua University, Beijing, China

**Keywords:** Intestinal stem cell, Stem cell niche, Telocyte, Trophocyte, Epithelial layer, Mesenchymal cell, Fibroblast, Stromal cell, Wnt, Lgr5, scRNA-seq

## Abstract

The rapidly self-renewing epithelium in the mammalian intestine is maintained by multipotent intestinal stem cells (ISCs) located at the bottom of the intestinal crypt that are interspersed with Paneth cells in the small intestine and Paneth-like cells in the colon. The ISC compartment is also closely associated with a sub-epithelial compartment that contains multiple types of mesenchymal stromal cells. With the advances in single cell and gene editing technologies, rapid progress has been made for the identification and characterization of the cellular components of the niche microenvironment that is essential for self-renewal and differentiation of ISCs. It has become increasingly clear that a heterogeneous population of mesenchymal cells as well as the Paneth cells collectively provide multiple secreted niche signals to promote ISC self-renewal. Here we review and summarize recent advances in the regulation of ISCs with a main focus on the definition of niche cells that sustain ISCs.

## Background

### Introduction: the intestine wall architecture and the intestinal epithelium

**Fig. 1:**
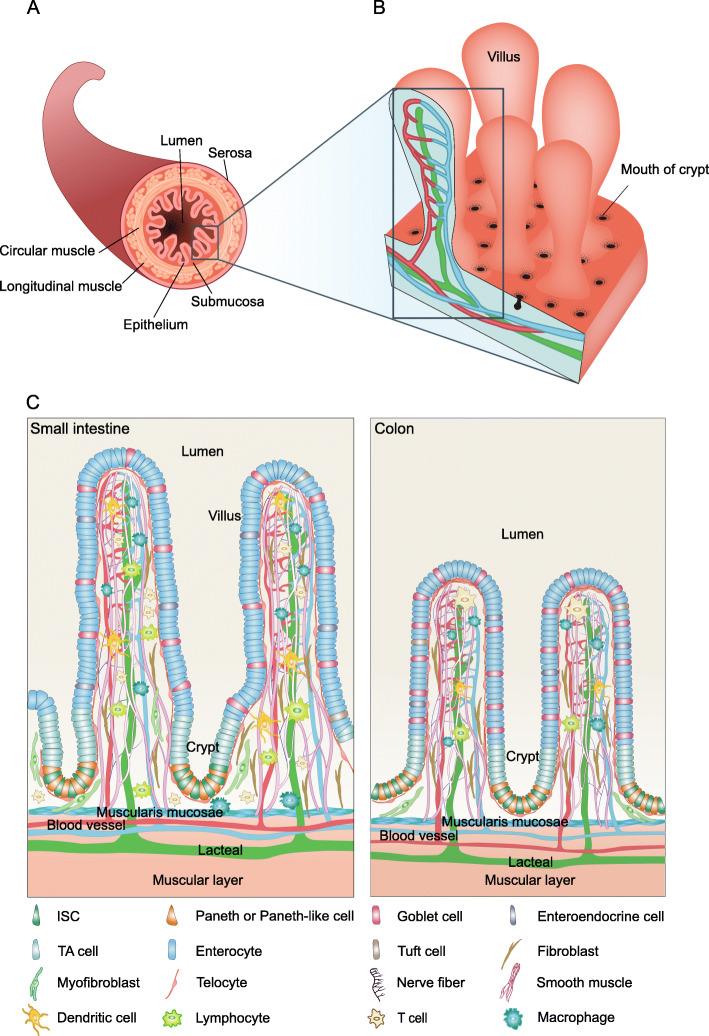
Anatomy of mammalian small intestine and colon. **a** A schematic showing the general tissue structure of the mammalian small intestine. **b** The enlarged view of the luminal surface of the small intestine. Each villus is surrounded by on average six crypts. **c** The enlarged view of the cellular architecture of the small intestine (left) and colon (right)

In mammals, the single-layered intestinal epithelium that lines the lumen of the small and large intestine performs important functions in food digestion and nutrient absorption, and at the same time it also serves as a front line of defense against pathogenic bacteria and toxins entered in the lumen (Fig. 1a). To maintain epithelial integrity, epithelial cells are continuously replaced by proliferating progenitors derived from multipotent intestinal stem cells (ISCs), with a turnover time of 3–5 days under normal conditions. The epithelium in the small intestine is organized into two morphologically and functionally distinct structures: the finger-like villi that protrude into the lumen, and the pocket-like crypts that harbor ISCs (Fig. 1b). Comparatively, the epithelium in the colon is composed of crypts that are relatively deeper and a relatively flat luminal surface with the absence of typical villi (Fig. 1c).

In addition to the epithelial layer, the intestine is also composed of three additional layers: lamina propria, a layer of loose connective tissue that contains a complex of cell populations including fibroblasts, pericytes and endothelial cells that constitute the vascular and lymphatic capillaries, and neural cell types, as well as scattered immune cells; circular and longitudinal smooth muscle layers; and serosa, the outermost layer enveloping the gut tube (Fig. 1c). Therefore, the bottom of the crypt where ISCs reside is surrounded by a complex microenvironment composed of diverse types of cells and membrane and capillary structures (Powell et al., 2011).

**Fig. 2:**
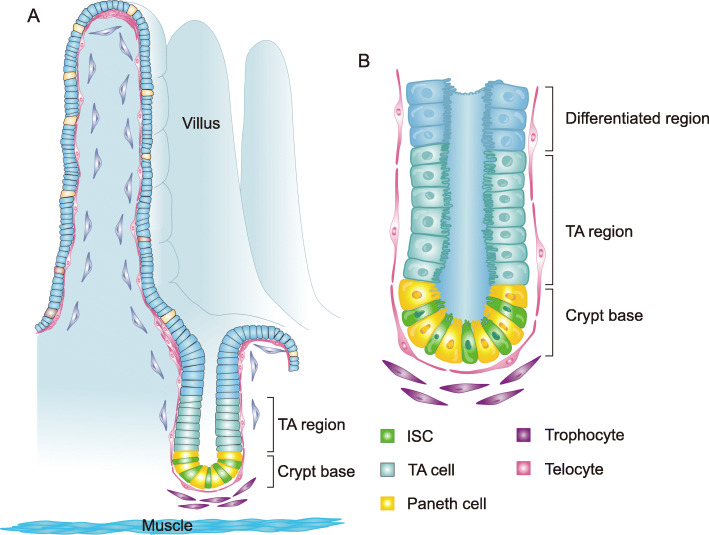
The ISC compartment and mesenchymal niche cells. **a** A schematic showing the cellular composition of the intestinal epithelium and the mesenchymal layer right beneath the epithelium. The ISCs are located at the bottom of crypt and are interspersed between Paneth cells. In the sub-epithelial compartment, the large flat telocytes form a sub-epithelial network to envelop the entire epithelium. The two clustered telocyte regions are found at the crypt-villus boundary and the villus tip. The trophocyte cluster is located at the pericryptal region, 2–3 cell diameters away from the pericryptal telocytes. **b** The enlarged view of the crypt

The ISCs in the small intestine reside at the bottom of the crypt and each crypt hosts 12–16 ISCs (Snippert et al., 2010). The ISCs are interspersed with a similar number of post-mitotic Paneth cells that collectively constitute a bowl-shaped stem cell compartment that occupies the crypt bottom. ISCs divide about once every 24 h. Once the ISCs leave away from the stem cell compartment, they quickly give rise to highly proliferative but short-lived progenitor cells in the transit amplifying (TA) region located in the remaining upper crypts (Fig. 2a). They go through 4–5 cell divisions before terminal differentiation towards various mature epithelial cell lineages including the secretory Paneth cells, goblet cells, enteroendocrine cells and tuft cells and the absorptive enterocytes. Most of them move upwards as differentiating to the flanks of intestinal villi to replenish the shedding epithelial cells at the top of villi, while the differentiated Paneth cells move back to the stem cell compartment to be interspersed with ISCs (van der Flier and Clevers, 2009) (Fig. 2a and b). In contrast with the small intestine, typical Paneth cells are absent from the mouse colonic epithelium, instead, a population of regenerating islet-derived family member 4 (Reg4)-positive deep crypt secretory (DCS) cells are found to be intermingled with colonic ISCs (Sasaki et al., 2016). Interestingly, typical Lyz1^+^ Paneth cells are found in colonic crypts in humans (Wang et al., 2020). Details on the regulation of differentiation of the ISC lineages can be found in several excellent reviews (Barker, 2014; Gerbe et al., 2012; van der Flier and Clevers, 2009; Yeung et al., 2011).

## Main Text

### Intestinal stem cells and facultative stem function of non-stem cells

Traditionally stem cells are defined by the capacity of self-renewal (for ISCs, generating more ISCs) and differentiation (for ISCs, giving rise to the entire mature lineages of the intestinal epithelium). Although historically ISCs had been long believed to reside at the bottom of the crypt (Crosnier et al., 2006), the precise location and identity of ISCs remained obscure until 2007, when Clevers and colleagues used a set of cell lineage tracing experiments to convincingly demonstrate that the crypt base columnar (CBC) cells marked by the expression of a Wnt target gene, leucine-rich repeat containing G protein-coupled receptor 5 (Lgr5), are actively cycling, long-lived and multipotent ISCs that maintain intestinal homeostasis through continuous generation of progenitor cells that differentiate into all the cell lineages found in the intestinal epithelium (Barker et al., 2007).

In addition to the active ISCs resided in the bottom of crypts, a quiescent reserve stem cell population located around the + 4 position, 4 cells distal to the bottom of the crypts, has been proposed, although these cells do not necessarily confer to the transitional view of stem cell definition. Several independent studies have identified this type of cells using different cell markers, such as Bmi1 (BMI1 proto-oncogene, polycomb ring finger), Hopx (HOP homeobox) or mTert (Mouse telomerase reverse transcriptase)(Sangiorgi and Capecchi, 2008; Takeda et al., 2011; Tian et al., 2011; Yan et al., 2012). Cell lineage tracing experiments suggest that they normally do not perform stem cell function, but can give rise to Lgr5^+^ ISCs under severe injury conditions such as radiation treatment, which causes the loss of Lgr5^+^ ISCs.

Although the active ISCs and reserve ISCs have been initially considered to be mutually exclusive (Li et al., 2014; Yan et al., 2012), a later study reveals the robust expression of the proposed reserve ISC markers Bmi1, Hopx, Tert and Lrig1, in Lgr5^+^ ISCs (Muñoz et al., 2012). Several follow-up studies on the process of radiation-induced intestinal regeneration surprisingly find that the Lgr5^+^ ISCs are indispensable for intestinal regeneration and the reserve ISCs are also radiosensitive (Metcalfe et al., 2014). As the committed secretory progenitor cells are localized at around the + 4 position and are slow-cycling cells, the proposed quiescent reserve ISCs are likely the secretory progenitor cells that are capable of dedifferentiation into Lgr5^+^ ISCs under certain conditions (Buczacki et al., 2013). Consistent with this notion, it has been demonstrated that the Dll1^+^ (van Es et al., 2012b) or Atoh1^+^ secretory (Tomic et al., 2018) progenitor cells can be converted to Lgr5^+^ ISCs upon crypt damage. This dedifferentiation process is accompanied by dynamic reorganization of chromatin accessibility in progenitor cells (Jadhav et al., 2017).

In addition to the secretory progenitors, the highly proliferative and short-lived Alpi^+^ enterocyte progenitor cells can also regain stemness to replace the lost Lgr5^+^ ISCs (Jones and Dempsey, 2016; Tetteh et al., 2016). Moreover, the Lyz1^+^ Paneth cells are also able to acquire a stem cell-like transcriptome via Notch and SCF/c-Kit signaling to contribute to epithelial regeneration following intestinal inflammation (Schmitt et al., 2018; Yu et al., 2018), and differentiated enteroendocrine cells can also revert to Lgr5^+^ ISCs and regenerate the intestinal epithelium after ISC loss (Yan et al., 2017).

Interestingly, single cell analysis of the ISC lineage conducted in a recent study reveals a damaged-inducible population of slow-cycling ISCs named as revival stem cells. These Clusterin (Clu)^+^ revival ISCs are multipotent, but they are rare and have very limited contribution to normal intestinal homeostasis. However, they are able to rapidly expand and regenerate the intestinal epithelium upon epithelial injury by irradiation (Ayyaz et al., 2019), indicating that the revival ISCs represent a separate population of multipotent ISCs that primarily function to restore the active ISCs following damage. However, using multiple sets of cell-labeling tools and genetic analyses, a recent study demonstrates that, following genetic ablation of Lgr5^+^ ISCs, the regeneration of intestinal epithelium is fueled mainly by ISCs dedifferentiated from the progenies of the Lgr5^+^ ISCs. The dedifferentiation process requires the activation of a transcription factor Ascl2, whose expression is normally restricted to ISCs, where it regulates the competitive fitness of ISCs in the niche compartment (Murata et al., 2020).

Taken together, those observations suggest that many non-stem cells in the ISC lineage are highly plastic, and can be converted to ISCs when the resident ISCs are lost. This cellular plasticity of the differentiated ISC progeny provides a robust mechanism for safeguarding ISC pools and thereby maintaining the regenerative ability of the intestinal epithelium. The broadly permissive chromatin structure found in both ISCs and differentiating progenitor cells may underlie the cellular plasticity of the intestinal cells (Kim et al., 2014).

### The regulation of ISCs

The self-renewal and differentiation of ISCs are regulated by an intricate interplay among extrinsic niche signals including Wnt, Notch, BMP and EGF signals and intrinsic transcription factors, chromatin regulators, and metabolic pathways. Transcription factors such as TCF/β-catenin, Ascl2 (Schuijers et al., 2015), Plagl2 (Strubberg et al., 2018), Gata6 (Whissell et al., 2014), Myb (Cheasley et al., 2011), Cdx2 (Simmini et al., 2014), and Yy1 (Perekatt et al., 2014), directly promote expression of ISC-enriched genes to maintain ISC fate. Overlapping and/or antagonistic activities of chromatin modifiers Dnmt1 (Elliott et al., 2015), Dnmt3b (Elliott et al., 2016), and Tet1 (Kim et al., 2016) are important for maintaining proper DNA methylation levels on ISC signature genes, whereas H3K27me3 modification on those genes are regulated by the cooperative activity among PRC1 (Chiacchiera et al., 2016a), PRC2 (Chiacchiera et al., 2016b), and HDAC (Cao et al., 2015). The long noncoding RNA LncGata6 has a role in ISC maintenance by recruiting the NURF complex to activate Ehf expression, which then induces Lgr4/5 expression to enhance Wnt signaling (Zhu et al., 2018). Lastly, the increased mitochondrial oxidative phosphorylation (OXPHOS) in the ISCs is also important for the maintaining of their stem cell function (Rodriguez-Colman et al., 2017). In contrast, mitochondrial pyruvate oxidation negatively regulates ISC proliferation (Schell et al., 2017).

As listed below, there are currently several major extracellular-to-intracellular signaling pathways considered to be essential for ISC self-renewal. The identification of the cell source of those secreted signals is therefore critical for the definition of the niche cells that sustain ISCs.

#### Wnt/β-catenin and R-spondins

The Wnt/β-catenin signaling is a major signaling transduction pathway used in ISCs for their self-renewal (Clevers et al., 2014). The pathway is evolutionarily conserved in metazoans and the components of the signaling cascades have been well-defined based on the study from various systems and genetic organisms, especially *Drosophila* (Clevers, 2006; Moon et al., 2002; van Amerongen and Nusse, 2009). Briefly, the Secreted Wnt signals bind to the frizzled receptors and LRP5/6 co-receptors, which leads to inhibition of adenomatous polyposis coli (*Apc*)- dependent ubiquitination of β-catenin (Kuhnert et al., 2004). β-catenin then accumulates in the cytoplasm and translocates to the nucleus where it interacts with the transcription factor TCF and other cofactors to activate the transcription of Wnt target genes.

R-spondins (Rspos), the ligands for the orphan receptors Lgr4/5/6, are considered as Wnt agonists as they can potentiate Wnt signaling. Rspo binding to the Lgr receptor enables Rspo to interact with E3 ligase RNF43/ZNRF3, which then promotes the endocytosis of the Rspo/Lgr complex. As RNF43/ZNRF3 is sequestered away from interacting with the Wnt/Fz/LRP complex, the Wnt/Fz/LRP complex persists on the plasma membrane, resulting in enhanced Wnt signaling strength and duration (de Lau et al., 2014).

The maintenance of ISCs is critically dependent on Wnt signaling activity. Conditional knockout of TCF4, β-catenin, transgenic expression of Wnt inhibitor Dickkopf 1 (Dkk1) or deletion of Rspo receptors Lgr4/5 all lead to the rapid loss of proliferating crypt (Fevr et al., 2007; Ireland et al., 2004; Kuhnert et al., 2004; Pinto et al., 2003; van Es et al., 2012a). Conversely, transgenic expression and injection of the Wnt agonist Rspo1 cause hyper-proliferation of crypt cells (Kim et al., 2005). The majority of colorectal cancers carry *Apc* mutations that result in Wnt pathway overactivation. In relatively rare cases, the cancers carry oncogenic point mutations in β-catenin (Kinzler and Vogelstein, 1996; Liu et al., 2000; Morin et al., 1997). Gene fusions that induce stronger expression of Rspos have also been found in human colorectal cancers (Seshagiri et al., 2012). Mutations in Rnf43 are also found in some human colon cancer cell lines (Koo et al., 2012). Rspo1 is also a critical exogenous factor in the intestinal organoid culture system, which maintains self-renewal and differentiation capacity of ISCs in vitro (Sato et al., 2009).

Comparative gene expression analysis of colorectal cancer cell lines, human adenomas and adenocarcinomas as well as normal colonic epithelium has revealed 121 genes that are potential Wnt/TCF target genes. In situ hybridization experiment further confirms 17 genes that are specifically expressed in the crypt ISCs (Van der Flier et al., 2007), which include the ISC marker Lgr5, the transcription factor Ascl2 which contributes to ISC proliferation, and the E3 ligase ZNRF3, which regulates the endocytosis of the Wnt receptor complex Fz/LRP, thereby establishing a negative feedback loop to control Wnt signaling activity (Koo et al., 2012).

#### Notch

Notch signaling is another evolutionarily conserved cell-to-cell signaling cascades that is initially characterized in *Drosophila* (Lai, 2004). In mammals, the transduction of Notch signaling starts with the binding of the Notch ligands Jagged (Jag- 1 and 2), and Delta-like (Dll- 1, 3 and 4) to the receptors (Notch 1–4). This leads to the activation of Notch by proteolytic cleavages that generate Notch intracellular domain (NICD). Subsequently, NICD translocates to the nucleus and interacts with DNA-binding transcription factor RBP-J to activate transcription of target genes (Kopan and Ilagan, 2009).

Notch signaling is utilized in the mammalian ISC compartment for maintaining the stemness of ISCs. The Paneth cells produce and secrete the Notch ligand Dll1 and Dll4 to activate Notch in the neighboring ISCs, which predominantly express Notch 1 and Notch 2 receptors (Pellegrinet et al., 2011; Sato et al., 2011). Inhibition of Notch activity leads to ISC loss and secretory cell hyperplasia, whereas overactivation of Notch signaling causes the expansion of intestinal progenitor cells (Carulli et al., 2015; Fre et al., 2005). In addition to enhancer of split family genes, *Olfm4* is also considered as a transcriptional target of Notch in ISCs (VanDussen et al., 2012).

#### Bone morphogenetic protein (BMP)

Opposite to the function of Wnt signaling, BMP signaling functions to inhibit ISC proliferation and promote ISC differentiation. The activities of BMP and Wnt signaling form reverse gradients along the crypt-villus axis to orchestrate self-renewal and differentiation of ISCs. Mechanistically, BMP signaling is found to negatively regulate the stemness of ISCs by Smad-mediated transcriptional repression of a large number of Wnt signature genes in ISCs, such as Lgr5. This observation suggests that the stemness program of ISC is directly controlled by antagonistic activities of Wnt and BMP signaling (Qi et al., 2017). Disrupting BMP pathway activity in intestine causes ectopic crypt formation (BMP inhibition through transgenic expression of its antagonist Noggin) or intestinal polyposis (loss of BMP signaling through conditional inactivation of *Bmpr1a* in the epithelium) (Haramis et al., 2004; He et al., 2004; Qi et al., 2017). Mutation of the BMP pathway (*Bmpr1a*) has been found in human juvenile polyposis (Hardwick et al., 2008; Howe et al., 2001).

#### EGFR signaling pathways

The EGFR/ErbB signaling is widely used in various types of epithelial cells for cell proliferation, differentiation and migration. In intestine, the EGF ligand is expressed in Paneth cells and subepithelial mesenchymal cells (Sato et al., 2009), while EGFR is mainly expressed in ISCs and TA cells (Yang et al., 2017). The expression pattern of EGF and EGFR indicates that paracrine EGF signals induce EGFR activation in ISCs and TA cells to promote cell proliferation. Indeed, EGF serves as an important exogenous factor in the intestinal organoid culture system (Sato et al., 2009), in which inhibition of EGFR by an EGFR inhibitor Gefitinib or withdraw of EGF from the medium blocks ISC proliferation and induces them into quiescence (Basak et al., 2017). ISCs also specifically express Lrig1, a cell surface negative regulator of EGFR/ErbB (Laederich et al., 2004). Ablation of Lrig1 in ISCs leads to increased EGFR and ErbB expression and excessive ISC proliferation, leading to the development of intestinal adenomas (Powell et al., 2012; Wong et al., 2012). Thus, Lrig1 mediates a negative feedback mechanism to control ISC proliferation and intestinal homeostasis.

#### Hippo signaling pathway

The Hippo signaling pathway is best known for its role in cell growth and organ-size control (Mo et al., 2014; Zheng and Pan, 2019). In intestine of both flies and mice, inhibition of Hippo signaling is sufficient to cause epithelial hyperplasia, and similarly, forced activation of the downstream TEAD transcription factor YAP protein is sufficient to induce the expansion of undifferentiated progenitor cells (Cai et al., 2010; Camargo et al., 2007; Karpowicz et al., 2010). Deletion of YAP does not cause any visible intestinal defects under normal intestinal homeostasis, but it interferes with intestinal regeneration during DSS-induced colitis (Cai et al., 2010). This indicates that YAP is particularly important for intestinal regeneration in response to intestinal stresses. Surprisingly, prolonged activation of YAP during intestinal damage eventually causes the inhibition of Wnt signaling and consequently loss of crypts (Barry et al., 2013), indicating that differences in the duration and strength of YAP could lead to drastically different outcomes. The importance of YAP during damage repair can be also reflected by the observation that the appearance of Clu^+^ revival stem cells in the damaged intestine is dependent on Yap (Ayyaz et al., 2019).

#### Hedgehog signaling pathway

The Hedgehog (Hh) signaling pathway is an important mediator of the epithelium-mesenchyme crosstalk in both the developing and adult intestine. Paracrine Shh and Ihh ligands secreted from the epithelial cells activate the Hh signaling transduction pathway in the subepithelial mesenchymal cells and smooth muscle cells to regulate their proliferation and growth, which is important for the formation of the crypt-villus architecture and intestinal morphogenesis (Madison et al., 2005; Walton et al., 2012). In addition, the downstream transcription factor GLI2 also regulates the expression of Wnt and Rspo signals in the mesenchymal cells, which in return sustain the activity of ISCs and epithelial homeostasis (Coquenlorge et al., 2019).

### The intestinal stem cell niche

Studies of stem cells in diverse organs of many organisms have revealed a common principal of stem cell regulation, that is, adult stem cells usually reside in a specialized niche microenvironment, which provides necessary extracellular signals and substances to promote the long-term maintenance and self-renewal of stem cells (Morrison and Spradling, 2008). As mentioned earlier, the ISCs are interspersed with Paneth cells in the stem cell compartment right beneath the sub-epithelial compartment, which contains various types of mesenchymal cells, immune cells and others. The anatomical relationships indicate that the Paneth cells and cells in the sub-epithelial compartment could be important cellular components of the ISC niche (Fig. 1c). It turns out that the neighboring cells from the epithelium compartment and mesenchyme compartment both contribute to the production of niche signals that collectively promote ISC self-renewal and prevent their differentiation.

#### Paneth cell: the niche cell in the epithelial compartment

Paneth cells not only directly interact with ISCs, they also secrete Wnt3, EGF, and surface-bound Dll4 that respectively activate EGFR/Ras/MAPK, WNT and Notch signaling transduction pathways (Sato et al., 2009). The isolated Paneth cells are able to enhance ISC-derived intestinal organoid growth in vitro and can substitute the exogenous Wnt ligands (Sato et al., 2011). In addition, Paneth cells also enhance Igr5^+^ ISCs function by secreting lactate to sustain active mitochondrial oxidative phosphorylation which stimulates p38/MAPK signal activation (Rodriguez-Colman et al., 2017). Therefore, Paneth cells provide multiple important niche signals that are known to be critical for ISC self-renewal. However, genetic confirmation of the niche function of Paneth cells is not straightforward, but rather surprisingly complicated.

An early study used a CR2-tox176 mouse model, which carries a transgene expression of diphtheria toxin driven by a Paneth cell specific cryptdin 2 promoter, to genetically ablate the Paneth cells, and found that this did not cause any detectable effect on ISC proliferation and crypt morphology (Garabedian et al., 1997). However, the Cre driver used in this study has a mosaic expression pattern that could cause incomplete ablation of Paneth cells (Sato et al., 2011). In another approach, the Paneth cells were ablated by conditional knock out of Sox9, a transcription factor that is essential for Paneth cell differentiation from ISCs (Bastide et al., 2007; Mori-Akiyama et al., 2007). In this knock-out model, the loss of Paneth cells was found to be accompanied by the loss of ISCs (Sato et al., 2011). However, it remains possible that the loss of ISCs could be due to potential cell-autonomous function of Sox9 in ISCs.

Subsequently, several studies showed that the intestinal crypt architecture, Lgr5^+^ ISC function and Wnt signaling response were not disturbed although the entire loss of Paneth cells by conditional deletion of the transcription factor Math1 (Atoh1), an essential driver for intestinal secretory cell lineage commitment (Yang et al., 2001) (Durand et al., 2012; Kim et al., 2012).

In *Atoh1* knock-out mice, the Notch target gene Olfm4 remains to be expressed in ISCs. What is the source of Notch ligand? A recent study showed that following the ablation of Paneth cells, their positions were quickly occupied by other secretory cells, including enteroendocrine cells and tuft cells, which provide the ligands for Notch activation in ISCs (van Es et al., 2019). Therefore, the source of Notch ligand could be provided by other secretory cells or secretory progenitor cells in case the Paneth cells are failed to form or eliminated.

These observations collectively suggest that the Paneth cell niche is not absolutely required for ISC self-renewal and indicate that other cellular sources of niche factors contribute to maintaining the stemness of ISCs in intestine. Although Paneth cells appear to be dispensable for normal intestinal homeostasis, they are important for stress-induced intestinal regeneration. Lyz1^+^ Paneth cells contribute to inflammation response and can dedifferentiate to Lgr5^+^ ISCs during injury-induced regeneration (Schmitt et al., 2018; Yu et al., 2018). The damaged intestines show powerful recovery ability. However, without the presence of Paneth cells, this repair ability is greatly compromised and the damaged intestines show a collapse of epithelial architecture (Parry et al., 2013). Therefore, although Paneth cells are not absolutely required for ISC maintenance, they provide important niche signals such as Wnt, Dll and EGF that contribute to ISC self-renewal, especially in stress-induced regeneration conditions when the niche signals are in high demand. The Reg4^+^ or cKit^+^ DCS cells in colon is functionally equivalent to Paneth cells in small intestine (Rothenberg et al., 2012; Sasaki et al., 2016), but they do not express Wnt ligands (Sasaki et al., 2016). The niche cell types in the epithelial compartment and their secreted niche signals are summarized in Table 1.

**Table 1:** The cellular orign of niche signals in the epithelial compartment

Region	Cell type	Produced signals	Function	Methods	Ref.
Small intestine	Paneth cells	Wnt3; Notch ligand (Dll4); Growth factors (TGFα and EGF);	Promoting ISC self-renewal	The isolated Paneth cells can replace Wnt3 to support intestinal organoid growth in 3D cultures;	(Sato et al., 2011)
				Mixed results about the requirement for ISC maintenance in vivo.	(Bastide et al., 2007; Durand et al., 2012; Emily M. Garabedian, 1997; Kim et al., 2012; Mori-Akiyama et al., 2007; Sato et al., 2011; van Es et al., 2019)
	Enteroendocrine cells (facultative)	Dll1	Can replace the vacated positions following Paneth cell ablation to support ISC maintenance.	Following the ablation of Paneth cells in Lyz1-DTR mice, EEs and tuft cells can repopulate the vacancies and provide Notch ligands for maintaining ISCs.	(van Es et al., 2019)
	Tuft cells (facultative)	Dll1	Same as above.	Same as above	(van Es et al., 2019)
Large intestine	Reg4^+^ deep secretory cells (DSCs)	Dll1 and Dll4; EGF	Maintenance of colonic ISCs	Ablation of DSCs in *Reg4*^*DTR-Red/+*^ mice causes ISC loss; Coculturing of ISCs with Reg4^+^ DSCs support organoid formation from single ISCs.	(Rothenberg et al., 2012; Sasaki et al., 2016)

#### Mesenchymal cells: the niche cells in the subepithelial compartment

It has been well-established that the canonical Wnt/β-catenin signaling cascade is absolutely required for the self-renewal of ISCs (Clevers et al., 2014). As Wnt3-producing Paneth cells are dispensable for ISC self-renewal, there must be additional sources of Wnts from the sub-epithelial mesenchyme that act on ISCs. Consistent with this notion, blocking Wnt secretion in the epithelial compartment alone by epithelium-specific gene knock-out of Porcupine (Porcn) or Wntless (Wls) has no obvious defects in intestinal homeostasis (Farin et al., 2012; Kabiri et al., 2014; San Roman et al., 2014), but blocking Wnt secretion globally in both epithelial and sub-epithelial compartment of the intestine leads to ISC loss (Valenta et al., 2016). Indeed, several Wnt ligands, such as the canonical Wnt2b, and non-canonical Wnt4, Wnt5a andWnt5b, are found to be expressed in the mesenchymal cells in intestine (Gregorieff et al., 2005). In addition, the Wnt signaling agonists Rspo proteins are also found to be expressed in the mesenchymal cells (Kabiri et al., 2014).

The mesenchymal cells are heterozygous, and it is important to determine whether the niche function is exerted by specific subsets of mesenchymal cells. Facilitated by advances in single cell technology over the past several years, rapid progress has been made in characterizing the mesenchymal subtypes in respect to their roles in regulating ISCs. Listed below are several representative mesenchymal subtypes that have been identified by specific expression of certain gene markers, and their contributions to ISC regulation have been studied in vivo and/or in 3D organoid culture conditions. These different groups of mesenchymal cell populations are not necessarily mutually-exclusive to each other, and as a matter of fact, some populations show extensive overlap with each other. This issue will be discussed at the end of the session.

##### Foxl1^+^ mesenchymal cells (telocytes)

A population of mesenchymal cells marked by winged-helix transcription factor forkhead box L1 (Foxl1) is the first mesenchymal cell subtype that has been demonstrated to be a critical component of the intestinal stem cell niche. Foxl1 (also known as Fkh6) was initially found to be expressed in the mesenchymal cells in the mouse fetal gut that are in close proximity to the epithelium, and the *Foxl1* knock-out mice show dysregulation of epithelial proliferation in the small intestine (Kaestner et al., 1997). These observations suggest that Foxl1 may regulate signals that mediate communications between the mesenchyme and the epithelium of the gut to regulate ISC proliferation. Subsequently, the Kaestner’s group further investigated the function of Foxl1^+^ mesenchymal cells by diphtheria toxin-induced cell ablation experiments in transgenic *Foxl1-hDTR* (a BAC clone of human diphtheria toxin receptor gene driven by the Foxl1 promoter) and *Foxl1-Cre;Rosa-iDTR* (Rosa–inducible DTR) mouse models. Ablation of Foxl1^+^ by either approach caused rapid cessation of epithelial proliferation and diminished Wnt signaling activity in the ISC and TA zones in the epithelial compartment (Aoki et al., 2016). Bulk gene expression analysis of Foxl1^+^ cells reveals the expression of multiple niche signal molecules, including Wnt2b, Wnt5a, Rspo3 and BMP inhibitors chordin-like1 and Gremlin (Grem) 1/2 (Aoki et al., 2016), indicating that the Foxl1^+^ mesenchymal cells produce multiple niche signals required for the self-renewal of ISCs. However, Foxl1^+^ mesenchymal cells also express the Wnt inhibitors Dkk3 and sFRP1 and Bmp4/5/6/7(Shoshkes-Carmel et al., 2018). This raises a concern about whether Foxl1^+^ mesenchymal cells provide an indispensable source of Wnt-active and BMP-inhibitory signals for ISC self-renewal.

In a subsequent study, the Kaestner’s group conducted experiments to specifically block the Wnt secretion function of Foxl1^+^ cells to address this issue. They found that the conditional knockout the Porcn gene in Foxl1^+^ cells caused a dramatic reduction of cell proliferation in the ISC and TA regions of small intestine and colon as soon as 3 days after tamoxifen administration (Shoshkes-Carmel et al., 2018). Therefore, the Foxl1^+^ mesenchymal cells are important Wnt-producing niche cells that sustain ISCs. These Foxl1^+^ mesenchymal cells are rare in adult intestine and belong to a subset of PDGFα^+^ cells, and labeling these cells with membrane-tethered GFP reveals that these cells are large flat cells distributed right beneath the epithelium in both crypt and villus regions and formed a thin layer of plexus surrounding the entire epithelium. Because these cells can send long extensions to connect with each other, the authors named these cells as “telocytes”, a name previously used to describe a type of stromal cells found in many organs that is characterized by extremely long and thin protrusions (Cretoiu and Popescu, 2014; Pellegrini and Popescu, 2011).

##### Gli1^+^ mesenchymal cells

During intestine development, Hh signals from the endodermal epithelial cells transverse through the basement membrane and act on the subepithelial mesenchymal cells to promote cell proliferation (Madison et al., 2005). Gli1, a transcription factor that mediates Hh signaling, is found to be expressed in a subset of mesenchymal cells right beneath the epithelium, a pattern that is similar to Foxl1^+^ cells (Degirmenci et al., 2018; Shoshkes-Carmel et al., 2018). When the *wls* gene is conditionally knocked out in these Gli1^+^ cells in [*Gli1-cre*^*ERT2*^*; Wls fl/fl*] mice, the ISCs in colon will be lost in 2 weeks. However, the ISCs in small intestine remain largely normal. If *wls* is simultaneously knocked out in epithelial cells as well with the addition of *villin-cre*^*ERT2*^ transgene, the ISCs in the small intestine will be lost as well (Degirmenci et al., 2018). Therefore, Gli1^+^ cells are essential Wnt producing cells for ISC maintenance in colon, but in small intestine, Gli1^+^ cells and epithelial cells collectively provide redundant Wnt signals for ISC maintenance.

Single cell analysis of the Gli1^+^ cells reveals 8 subclusters with CD34^+^ subclusters that express abundant Wnt signals, including Wnt2, Wnt2b, and Wnt4. Two other subclusters are Foxl1^+^, suggesting that Gli1^+^ and Foxl1^+^ cell populations partially overlap with each other (Degirmenci et al., 2018).

##### CD34^+^ mesenchymal cells

As mentioned above, a subset of Gli1^+^ cells are CD34^+^ cells that express multiple Wnt ligands. An earlier study shows that the CD34^+^GP38^+^ mesenchymal cells, a subset of CD34^+^ population, can specifically mark pericryptal cells, the mesenchymal cells surrounding the crypt region in adult small intestine and colon (Stzepourginski et al., 2017). Bulk RNA-sequencing analysis reveals the expression of Wnt2b in this cell population in normal intestine, and a dramatic upregulation of Rspo1 and Grem1 in this population following the treatment of Dextran Sodium Sulfate (DSS), which causes intestinal inflammation. In cultured organoids from ISCs, the addition of these cells to the culture causes the organoids with budding crypts to become spherically-shaped organoids, a phenotype due to increased ISC proliferation and impaired epithelial differentiation (Stzepourginski et al., 2017). Collectively, these observations suggest that the pericryptal CD34^+^ cells may have important functions in regulating ISC proliferation during both normal homeostasis and during intestinal repair after injury.

##### Ng2 (Cspg4) ^+^ pericyte-like cells

By single-cell analysis of the stomach and small intestine, Kim and his colleagues find that Cspg4 (or Ng2) not only marks the pericyte population, but also marks many non-pericyte cells that show extensive overlap with Foxl^+^ populations in the mesenchyme of both stomach and small intestine (Kim et al., 2020). RT-PCR analysis of these Ng2^+^ perictye-like mesenchymal cells reveals the expression of Wnt2b and Wnt4. Blocking Wnt secretion in these perictye-likes in [*Ng2-cre; Wls fl/fl*] mice causes the reduction of ISC number, but the overall proliferative landscape of the epithelium remains largely normal (Kim et al., 2020). This phenotype is much weaker than that caused by blocking Wnt secretion in Foxl1^+^ cells, indicating that the Ng2^+^ mesenchymal cell population does not contain all Foxl1^+^ mesenchymal cells.

##### CD90^+^ mesenchymal cells (Colon)

In mouse colon, a subpopulation of Gli1^+^ gp38^+^ cells specifically marked by CD90 is found to be located at the bottom of the crypt enveloping the proliferating ISC and TA cells, a distribution pattern similar to CD34^+^ cells. Microarray analysis of these CD90^+^ cells show the expression of multiple niche factors including Rspo3, Wnt2b and Grem1 as well as a family of semaphorins that previously known to have a role in axon guidance. These semaphorins appear to have a role in regulating ISC proliferation, as inhibiting the function of the Nrp2 receptor blocks the growth-promoting effect by CD90^+^ cells on the cultured colonic organoids (Karpus et al., 2019).

##### CD81^+^ trophocytes

PDGFRα^+^ mesenchymal cells represent a relatively large population of mesenchymal cells in the sub-epithelial compartment. Conditional knockout of *Porcn* specifically in this cell population in the small intestine impairs crypt formation (Greicius et al., 2018), revealing that the PDGFRα^+^ mesenchymal cells provide Wnt signals necessary for ISC maintenance. In addition to Wnts, the PDGFRα^+^ mesenchymal cells also express Rspo3, which is more potent than Rspo1 in promoting organoid growth. In contrast to *Porcn*, mice with conditional *Rspo3* knockout in PDGFRα^+^ cells do not display any perturbation in intestinal homeostasis. However, these mice show increased sensitivity to DSS treatment, reflected by severe crypt loss and intestinal inflammation (Greicius et al., 2018), suggesting that Rspo3 derived from the mesenchymal cells is not required for normal intestinal homeostasis but is essential for the repair of the epithelium after tissue damage.

Based on single-cell RNA-sequencing analysis, the PDGFRα^+^ mesenchymal population can be divided into two subpopulations, PDGFRα^high^ and PDGFRα^low^ subpopulations. The Foxl1^+^ telocyte population is inclusive to the PDGFRα^high^ population. The PDGFRα^low^ subpopulation can be further divided into two subtypes, with one of them expresses Grem1 and can be specifically marked by CD81. Remarkably, the isolated CD81^+^ cells can support organoid growth alone without adding trophic factors to the culture medium. These CD81^+^ cells are thus renamed as trophocytes (McCarthy et al., 2020). Comparatively, the isolated PDGFRα^low^ CD81^−^ cells do not support organoid growth unless the BMP inhibitor Noggin is exogenously added to the culture. Therefore, the BMPi activity is a major mechanism underlying the organoid growth-promoting function of trophocytes. In situ hybridization of Grem1, which marks the trophocytes, shows that the trophocytes predominately reside below crypts, only a few cell diameters away from the ISC compartment. Ablation of these trophocytes in [*Grem1-creERT2; Rosa26-DTR*] mice (the surrounding smooth muscle cells are also ablated as they are also Grem1+) causes loss of ISCs in the crypt within 2 days of Grem1^+^ cell ablation (McCarthy et al., 2020). Collectively, these observations suggest that the Grem1^+^ trophocytes below the crypt base constitute the ISC niche at least by providing BMPi environment required for ISC maintenance. As mice deficient for Grem1 do not have obvious defects in intestinal homeostasis, the trophocytes should have other important functions in addition to providing Grem1. Not surprisingly, these cells also express multiple Wnts and Rspos (McCarthy et al., 2020), and therefore could serve as an important source for Wnt-promoting activity as well.

##### Other cell types in the mesenchymal compartment

*Immune cells.* Tissue-resident innate immune cells such as innate lymphoid cells (ILCs) have also recently been implicated in regulating intestinal regeneration. For exmaple, group 2 ILCs (ILC2s) secrete IL-13 that is able to activate IL-13/IL-13R signaling in ISCs, which contributes to the maintenance of ISCs (Zhu et al., 2019). Following intestinal damage, ILCs also secrete IL-22 signal to ISCs and activate STAT3 to promote ISC proliferation and epithelial regeneration (Lindemans et al., 2015). The adaptive immune cells could also be modulators of ISCs, such as regulatory T cells, which interact with ISCs to promote ISC renewal and epithelial remodelling via secreting IL-10 following intestinal infection (Biton et al., 2018).

*Endothelial cells.* Gene expression analysis reveals the expression of some Wnt-related niche signals in the endothelial cells (Table 2). In addition, an early study shows that radiation-induced crypt damage can be prevented when endothelial apoptosis was inhibited, raising a possibility that the apoptotic endothelial cells could send some signals to induce ISC dysfunction following radiation damage (Paris et al., 2001). The signals from the apoptotic endothelial cells remain unknown, but metabolites released from apoptotic cells could act as messengers (Medina et al., 2020).

**Table 2:** The cellular origin of ISC niche signals in the mesenchymal compartment

Cell type	Produced signals	Function	Methods	Ref.
Foxl1^+^ telocytes	Wnt-act (Wnt2b, 5a, Rspo3); Wnti (Sfrp1, Dkk3); BMPi (Chrdl1, Grem 1); BMPs (BMP4, 5, 6, 7); Others (Fgf7, Hgf, Igf1, Igfbp5, Pgf, Ctgf)	As an essential source of Wnt signals for ISCs.	Ablation of Foxl1+ cells in *Foxl1-hDTR* or *Foxl1-cre; Rosa26-iDTR* mice causes abrupt cessation of ISC proliferation; Deletion of Porcn in *Foxl1-creERT2* mice causes rapid decline of Wnt activity and crypt loss.	(Aoki et al., 2016; Shoshkes-Carmel et al., 2018)
Gli1^+^ mesenchymal cells	Wnt-act (Wnt2, 2b, and 4; Rspo3); Wnti (Sfrp1)	An essential Wnt source in colon and a reserve Wnt source in SI; Responsive to damage-induced intestinal regeneration	Deletion of Wls in *Gli1-creERT2* mice causes ISC loss in colon over time; Deletion of Wls in *Vilin-creERT2* and *GLI1-creERT2* combined mice causes reduced ISC proliferation in SI over time; ScRNA-seq data suggests the expansion of Gli^+^ cells following epithelial damage.	(Degirmenci et al., 2018)
CD81^+^PDGFR-α^lo^ trophocytes	BMPi (Grem1)	Sustain ISCs in vivo; promote ISC expansion in vitro.	Ablation of Grem1^+^ cells in *Grem1–creERT2; Rosa26–iDTR* mice causes rapid ISC loss; The Grem1^+^ trophocytes can support enteroid growth without exogenous Wnt/Rspo and BMPi factors.	(McCarthy et al., 2020)
CD34^+^GP38^+^ mesenchymal cells	Wnt-act (Wnt2b, Rspo1); BMPi (Grem1); Others (Areg, Fgf7, Fgf10, Ptgs2 and Col1a1);	Maintain ISCs in vitro; contribute to intestinal repair after injury.	CD34^+^Gp38^+^ cells promote ISC maintenance in intestinal organoids; these cells are rapidly expanded in DSS-mediated colitis.	(Stzepourginski et al., 2017)
PDGFR-α^+^ pericryptal stromal cells	Wnts and Rspo3	As critical source of Wnts and Rspo3 for ISC self-renewal.	Deletion of Porcn in *PdgfRα-cre* mice blocks crypt formation; Deletion of Rspo3 in *PdgfRα-cre* mice causes decreased crypt WNT/β-catenin signaling and predisposes to DSS-induced colitis.	(Greicius et al., 2018)
Cspg4^+^ pericyte-like	Wnt2b and Wnt4	Required for gut regeneration.	Deletion of Wls using *Cspg4-cre* mice causes reduced ISCs and comprised regeneration following irradiation.	(Kim et al., 2020)
CD90^+^ fibroblasts	BMPi (Grem1); Wnt-act (Wnt2b, and Rspo3); Others (Sema3).	Support the organoid growth.	Co-culture of CD90^+^ crypt fibroblasts supports organoid growth in R-spondin-reduced medium;	(Karpus et al., 2019)
Myofibroblasts	Wnt-act (Rspo3); Wnti (Dkk3);	Uncertain	Coculture of myofibroblasts and SI crypts supports long-term growth without Rspo1.	(Lei et al., 2014)
			Deletion of Porcn using *Myh11-creERT2* and/or *Villin-creERT2* lines all fails to disrupt crypt proliferation or Wnt pathway activity;	(San Roman et al., 2014)
Endothelial, immune, smooth muscle cells, macrophages, etc.	Wnt-act (Rspo3), Wnti, BMPi and NOTCH ligands (Dll1 and Dll4) in many of these cells	N. D	N. D.	(Bauché et al., 2018; Hansen et al., 2019; Ogasawara et al., 2018)

##### Summary of mesenchymal cell subtypes and function

**Fig. 3:**
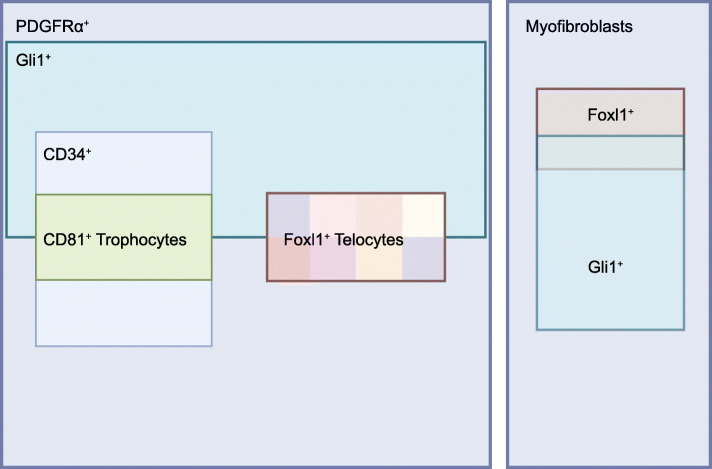
Inclusion relationships among various subtypes of mesenchymal niche cells. Each rectangle represents a specific population of mesenchymal cells as indicated and is color-coded. Note: the telocyte population contains both peri-crypt and peri-villus telocytes and is likely heterogeneous; some myofibroblasts also express the telocyte markers Foxl1 and Gli1

The niche cell types in the sub-epithelial compartment and their secreted niche signals are summarized in Table 2. As mentioned earlier, these different populations of mesenchymal cells characterized by various research groups are not necessarily mutually-exclusive to each other. Very likely, some of these populations could be largely identical or have extensive overlap with each other. Therefore, it is important to come up with a concerted view from these findings. Single cell analysis of the lamina propria in small intestine reveals that the PDGFRα cells cover all types of fibroblasts in the mesenchyme except myofibroblasts (Act2a2^+^ Myh11^+^) (McCarthy et al., 2020). The contribution of these myofibroblasts to ISCs appears to be very limited. Lei et al. show that the myofibroblasts can enhance the growth and differentiation of ISCs in vitro (Lei et al., 2014). However, Roman et al. demonstrate that blocking of Wnt secretion from subepithelial myofibroblasts has no effect on the proliferation and differentiation of ISCs (San Roman et al., 2014). In contrast, the essential role for the PDGFRα cells as the source of Wnt signals for ISC maintenance has been demonstrated genetically(Greicius et al., 2018). Therefore, the mesenchymal cells required for ISC self-renewal should be mainly within the PDGFRα^+^ populations. Single cell analysis of the PDGFRα^+^ population by McCarthy and colleagues reveals that CD34^+^ cells (express Wnt2b, Rspo1–3) and Foxl1^+^ (express non-canonical Wnts, Wnt4, 5a, 5b) are mutually-exclusive populations, and the CD81^+^ trophocytes belong to one of the two subpopulations of CD34^+^ cells. We have reanalyzed this single cell data and find that Gli1^+^ cells can be found in all the subpopulations of PDGFRα^+^, but appear not expressed in all PDGFRα^+^ cells, indicating that Gli1^+^ mesenchymal cells are extremely heterozygous and belong to a subset of PDGFRα^+^ cells (Fig. 3). Single cell analysis of the Gli1^+^ population by Degirmenci and colleagues shows similarly that the CD34^+^ subpopulations (express Wnt2, Wnt2b, Rspo3) and Foxl1^+^ subpopulations are mutually-exclusive, and also reveals that Gli1^+^ cells also contain myofibroblasts that are Foxl1^+^ (Degirmenci et al., 2018). Collectively, it appears that there are at least two distinct mesenchymal niche populations that are critical for ISC self-renewal: The CD34^+^ CD81^+^ trophocytes that provide BMPi (Grem1), Wnts (Wnt2b) and Rspo (Rspo1–3); and the Foxl1^+^ (or Cspg4/Ng2^+^) telocytes. In addition, the CD34^+^ CD81^−^ cells may contribute to the production of Wnt and Rspo signals. Based on those analyses, we have drawn a graph to reflect the proposed inclusion relationships among the above mentioned mesenchymal cell subtypes (Fig. 3).

The trophocytes reside in a relatively restricted region at the base of the crypt and appear to be a relatively homogenous population. The telocytes, however, resides broadly beneath the entire epithelium and appear to constitute a heterogeneous population of cells with distinct functions. Examination of the expression of several key niche signals in the telocytes reveals that these signals show compartmentalized pattern along the crypt-villus axis, with more Wnt2b and Rspo3 signals at the crypt base and increased Bmp5, Wnt5a and Dkk (Wnt inhibitor) signals towards the crypt-villus junction and villus tip (Shoshkes-Carmel et al., 2018). Therefore, the telocytes appear to be functionally compartmentalized along the crypt-villus axis which might contribute to the Wnt-BMP gradient formation along the crypt-villus axis that is known to be essential for self-renewal and differentiation of ISCs.

## Conclusions and future perspectives

Since the identification of the Lgr5^+^ ISCs in 2007, rapid progress has been made on the understanding of the niche microenvironment that sustains ISCs during both normal intestinal homeostasis and during epithelial repair following damage. It becomes increasingly clear that multiple stromal and epithelial cell populations collectively produce a diverse of niche signals to regulate ISC self-renewal and differentiation. The differentiated cells from the epithelial compartment, especially Paneth cells or Paneth-like cells, although are not essential for ISC maintenance and epithelial renewal during normal homeostasis, are important contributors of multiple niche signals and become critical for ISC maintenance during intestinal damage and repair. Remarkably, a heterogeneous population of stromal cells in the subepithelial compartment constitutes a key component of ISC niche as these cells are not only indispensable components of ISC niche during normal intestinal homeostasis by providing a diverse of niche trophic factors, they also appear to be able to expand themselves in response to intestinal damage to promote ISC activity and intestinal repair.

With the advances of single cell technology and easiness in genetic manipulations in mice, rapid progress has been made on the understanding of the heterogeneity of the subepithelial mesenchymal cells and two distinct subpopulations of mesenchymal cells have emerged and can now be considered as the key components of the ISC niche: the large flat telocytes that envelop the crypt epithelium, and the trophocyte clusters that reside at the crypt base and produce Grem1 and other trophic factors. Although the Foxl1^+^ (also Cspg4^+^) telocytes are relatively rare and usually grouped into a common cell cluster in the single cell analysis, this telocyte population envelops the entire intestinal epithelium and is likely heterogeneous. New studies are emerging that the telocytes that resides at the crypt, villus and inter crypt-villus regions could be potentially distinguished by different genetic markers (Halpern et al., 2019), and the development of new tools that can label the telocytes at specific regions should facilitate the investigation of the regional specific regulation and function of telocytes. Telocytes are characterized by their long cytoplasmic extensions, but it is unclear about the purpose of having those cytoplasmic extensions. In *Drosophila* testis, the germline stem cells send tube-like cytoplasmic structures to the neighboring hub for niche signaling reception (Inaba et al., 2015). Do the pericryptal telocytes mediate the transport of signals produced in the mesenchyme to the ISCs, and how? Are these cytoplasmic extensions able to traverse through the basement membrane and reach ISCs? How is the morphology of telocytes developed and maintained? These are all exciting outstanding questions about the mysterious telocytes that waits to be addressed.

Gene expression analysis of the telocytes reveals mixed expression of Wnt/BMP-promoting as well as Wnt/BMP-inhibiting signals, which makes it uncertain about their role in niche signal production and whether they provide a self-renewal-promoting or differentiation-promoting environment. This may also reflect the heterogeneous nature of the telocytes. In contrast, the CD34^+^ CD81^+^ trophocytes specifically express BMP antagonist Grem1, and this BMPi activity is also responsible for their growth-promoting activity found during organoid co-culture (McCarthy et al., 2020). Thus, the trophocytes constitute an essential component of the ISC niche by providing a BMPi environment to prevent ISC differentiation. It is worth noting that the BMPi function is unlikely the only function of trophocytes, as they are able to promote organoid growth without adding any trophic factors. Indeed, these cells as well as the CD34^+^ CD81^−^ cells also express Wnt and Rspo ligands, indicating that these cells may also contribute to the niche function by providing a Wnt-active environment. New tools are needed to allow specific genetic manipulation of trophocytes and CD34^+^ CD81^−^ cell populations so that their spatial distributions and biological functions can be further characterized and defined.

Although there are multiple different but related mesenchymal cells in the intestine, little is known about the mechanisms underlying the homeostasis of these mesenchymal cell populations. A related point is that it is unclear whether there are cell lineage relationships among them and whether there are stem cell populations that sustain these different mesenchymal subtypes. Better classification of the mesenchymal cell subtypes remains an important issue. As described earlier, many mesenchymal cell subpopulations that are marked by various markers may have significant overlaps among each other or may be very unspecific. For examples, the Foxl1^+^ cells and Cspg4^+^ cells might largely refer to a similar mesenchymal subpopulation, and the Gli1^+^ cells may contain a part of telocytes and a part of trophocytes, among others. A classification based on cell fate identity and cell lineage relationships would be ideal. This requires more comprehensive surveys of the mesenchymal cells at the single cell resolution, with the consideration of regional differences. The mesenchyme in the small intestine and colon clearly show differences, such as CD90, which only marks the peri-cryptal in colon but not small intestine. The mesenchyme in different regions of small intestine or colon could also be different. In addition, there may be significant differences in the mesenchymal populations between mice and humans (Kinchen et al., 2018). These issues should to be taken into consideration when investigating the mesenchymal cell lineages.

In addition to the mesenchymal cells in the subepithelial compartment, there are also immune cells that scattered distributed, endothelial cells and pericytes that constitute the lymphatic and vascular capillaries, and nerve cells. Many of these cells also express niche signals (Hansen et al., 2019), and their contribution to the regulation of ISCs and intestinal homeostasis awaits further investigation. The smooth muscle cells that surrounding the gut epithelium and mesenchyme express high levels of niche signals, such as Grem1 (McCarthy et al., 2020). The smooth muscle cells in the *Drosophila* intestine, which lacks the mesenchyme compartment, are known to serve as a regulatory niche for ISCs by secreting Wnts and EGFs that acts on ISCs (Lin et al., 2008; Xu et al., 2011). Although the muscle cells in the mammalian intestine are not in close proximity to the ISC compartment, it might be worthwhile to determine whether they could contribute to the regulation of ISCs by secreting niche signals that act over a long distance. Exampled by the newly defined telocytes and trophocytes, it is clear that much new and exciting biology has emerged from the subepithelial compartment in the regulation of ISCs and intestinal homeostasis. In addition, new studies are emerging to investigate the regulation and remodeling of these subepithelial cells in inflammatory bowel diseases and colorectal cancers (Kinchen et al., 2018; Parikh et al., 2019; Roulis et al., 2020; Smillie et al., 2019), and continuous understanding of the roles played by the mesenchymal niche cells may open new avenues for the prevention and treatment of many gastrointestinal diseases.
